# Medical transformer for multimodal survival prediction in intensive care: integration of imaging and non-imaging data

**DOI:** 10.1038/s41598-023-37835-1

**Published:** 2023-07-01

**Authors:** Firas Khader, Jakob Nikolas Kather, Gustav Müller-Franzes, Tianci Wang, Tianyu Han, Soroosh Tayebi Arasteh, Karim Hamesch, Keno Bressem, Christoph Haarburger, Johannes Stegmaier, Christiane Kuhl, Sven Nebelung, Daniel Truhn

**Affiliations:** 1 Department of Diagnostic and Interventional Radiology, University Hospital Aachen, Aachen, Germany; 2 Department of Medicine III, University Hospital Aachen, Aachen, Germany; 3 Else Kroener Fresenius Center for Digital Health, Medical Faculty Carl Gustav Carus, Technical University Dresden, Dresden, Germany; 4 Division of Pathology and Data Analytics, Leeds Institute of Medical Research at St James’s, University of Leeds, Leeds, UK; 5 Medical Oncology, National Center for Tumor Diseases (NCT), University Hospital Heidelberg, Heidelberg, Germany; 6 Physics of Molecular Imaging Systems, Experimental Molecular Imaging, RWTH Aachen University, Aachen, Germany; 7 Department of Radiology, Charité-University Medicine Berlin, Berlin, Germany; 8 Ocumeda GmbH, Munich, Germany; 9 Institute of Imaging and Computer Vision, RWTH Aachen University, Aachen, Germany

**Keywords:** Medical imaging, Radiography, Computer science

## Abstract

When clinicians assess the prognosis of patients in intensive care, they take imaging and non-imaging data into account. In contrast, many traditional machine learning models rely on only one of these modalities, limiting their potential in medical applications. This work proposes and evaluates a transformer-based neural network as a novel AI architecture that integrates multimodal patient data, i.e., imaging data (chest radiographs) and non-imaging data (clinical data). We evaluate the performance of our model in a retrospective study with 6,125 patients in intensive care. We show that the combined model (area under the receiver operating characteristic curve [AUROC] of 0.863) is superior to the radiographs-only model (AUROC = 0.811, p < 0.001) and the clinical data-only model (AUROC = 0.785, p < 0.001) when tasked with predicting in-hospital survival per patient. Furthermore, we demonstrate that our proposed model is robust in cases where not all (clinical) data points are available.

## Introduction

By definition, patients in intensive care are seriously and critically ill. In caring for those patients, intensive care provides a cornerstone of contemporary clinical medicine. Consequently, major hospitals usually operate at least one intensive care unit (ICU) to admit and treat those patients. Substantial financial resources that amount to about 1% of the gross domestic product in the United States are utilized annually to care for those patients^1^. These resources are used to improve patient monitoring and treatment. During the ICU stay, increasing amounts of clinical data are collected during patient diagnosis, treatment, and monitoring. Nowadays, most of these data are stored digitally and can be harvested from Electronic Health Records (EHR) systems and from picture archiving and communication systems (PACS) to be used in translational research^2^. Even with the advent of ever more powerful machine learning models, this plethora of data has not been used to the full extent. Machine learning models have predominantly used clinical data, i.e., EHR data^3–6^ or imaging data alone^7–10^. This approach contrasts with how physicians incorporate clinical data and patient information. Experts interpret imaging studies in clinical contexts to help distinguish between different disease states. Ideally, chest radiographs from the ICU should be interpreted with complete clinical data available to assess the patient’s state optimally, yet this may not always be the case. Combining expert knowledge from different specialties requires time-consuming consultations and may be challenging to realize on a 24/7 basis^11^. Accordingly, machine learning models that integrate non-imaging and imaging data are needed. Recent advances have seen the rise of transformer models that constitute the state-of-the-art technique in natural language processing and are applied to image processing with competitive performance as convolutional neural networks (CNNs)^12,13^.

Previous methods for predicting the survival of patients in intensive care have predominantly utilized combinations of CNNs and recurrent neural networks (RNNs)^14,15^. On the one hand, integrating non-imaging data into CNNs is challenging. It requires novel methods such as rescaling the feature maps^16^ or devising alternative means for presenting the non-imaging data in matrix form^17^. The latter approach means that the data are concatenated to the input image prior to feeding them into the neural network^17^. On the other hand, RNNs suffer from vanishing or exploding gradients, which limits the possible time horizon of extracted laboratory data^18^. Combining CNNs and RNNs necessitates a laborious multi-step approach. Modality-specific feature extractors are trained initially, followed by a fusion step combining the features for the final prediction^14^. In contrast, the transformer neural network is an input-agnostic method with a dedicated attention mechanism. A set of tokens is the only input, which may be easily created from various non-imaging and imaging data^12,13^. This approach enables end-to-end training and an intuitive combination of variable data sources. Imaging data-related tokens can now attend to non-imaging data-related tokens and vice versa. Furthermore, unlike RNNs, transformer neural networks do not rely on a long chain of sequential processing steps but on parallel processing, therefore mitigating the problem of vanishing and exploding gradients^13^.

To our best knowledge, transformer neural networks have not yet been used for survival predictions of patients in intensive care. The accurate prognosis is clinically relevant for these patients because (i) physicians may be better supported to decide if and how a patient may benefit from intensive care, and (ii) families may be better informed about the goals and potential advantages and disadvantages of intensive care.

This work presents the multimodal Medical Transformer (MeTra) that can process non-imaging and imaging data. Our architecture can learn from imaging data, non-imaging data, or a combination of both. We test our model on bedside chest radiographs, likely the most frequently ordered imaging study worldwide, accounting for approximately 20–25% of all diagnostic imaging activities in healthcare^19,20^. The accompanying non-imaging data (synonymous with clinical data and clinical parameters [CP]) to these radiographs represents the situation physicians encounter in the clinical routine. It comprises clinical tests (i.e., Glasgow Coma Scale), physiological parameters (i.e., heart rate, respiratory rate), blood serum parameters (i.e., glucose concentration, oxygen saturation), and information on body constitution (i.e., height and weight).

The overarching objective of this study was to apply and systematically evaluate the multimodal MeTra network architecture to integrate non-imaging and imaging data in the survival prediction of patients in intensive care, i.e., in the medical domain. We hypothesized that (i) the MeTra model would predict the survival of patients in intensive care more accurately when trained with imaging data, i.e., bedside chest radiographs, and non-imaging data, i.e., clinical data, than when trained with each data category alone. We also hypothesized that (ii) the MeTra model’s predictive performance would be robust and maintained when missing pertinent data.

## Results

### Characteristics of the dataset

**Figure 1:**
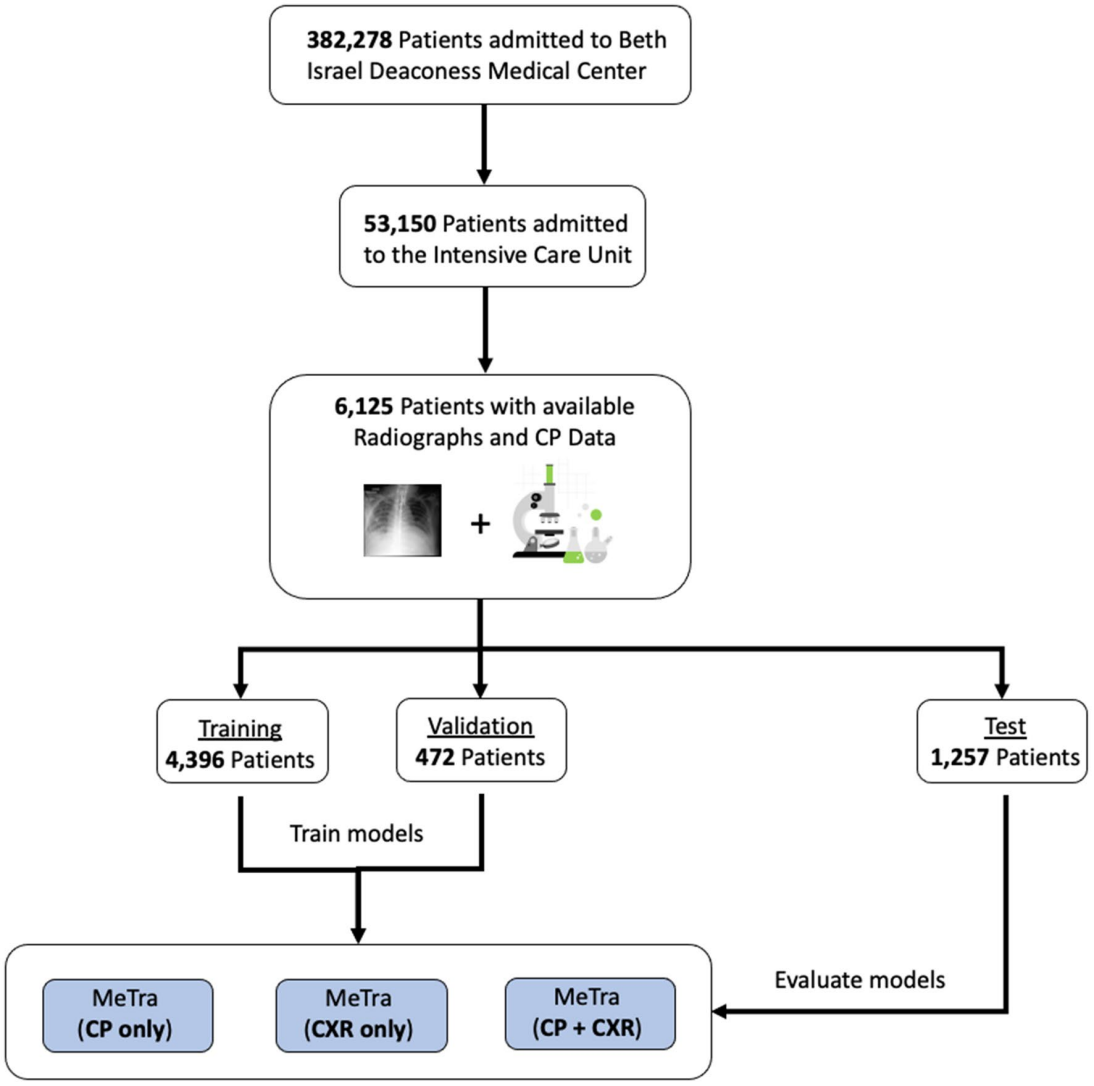
Visualization of the data extraction pipeline. For training, we only make use of those patients who were admitted to the Intensive Care Unit (n = 53,150) and who had clinical data (clinical parameters—CP) with matching chest radiographs available (n = 6,125). The data is split into the training (n = 4,396 patients), validation (n = 472 patients), and test sets (n = 1,257 patients).

Within the MIMIC-IV dataset^21^, 6125 patients had chest radiographs and clinical parameters, resulting in 6,798 bedside chest radiographs with corresponding clinical parameters (see Fig. 1). At the time of recording, patient age ranged from 18 to 91 years with a mean of 64 years ± 16 [standard deviation]. To preserve anonymity, all patients older than 89 years had been assigned the age of 91 years by the dataset providers. Of all patients, 55% (n = 3382) were male and 45% (n = 2743) were female. A total of n = 1,002 patients died in the hospital. A detailed description of the data is given in Table 1.

**Table 1:** Characteristics of the dataset.

Parameter	All patients	Training set	Validation set	Test set
No. of paired chest radiographs and clinical parameters	6798	4885	540	1373
No. of patients	6125	4396	472	1257
No. of male patients	3382 (55%)	2414 (55%)	264 (56%)	704 (56%)
Age (years) (range)	18—91	18—91	19—91	18—91
Mean age ± SD [Median] (years)	64 ± 16 [65]	64 ± 16 [65]	64 ± 16 [65]	64 ± 16 [65]
No. of deaths during hospital stay	1002 (16%)	717 (16%)	76 (16%)	209 (17%)

Data in parentheses are percentages. Note that the number (percentage) of deaths during the hospital stay for a given set is relative to the number of patients in each set.

### Results of MeTra model training on unimodal data only

**Figure 2:**
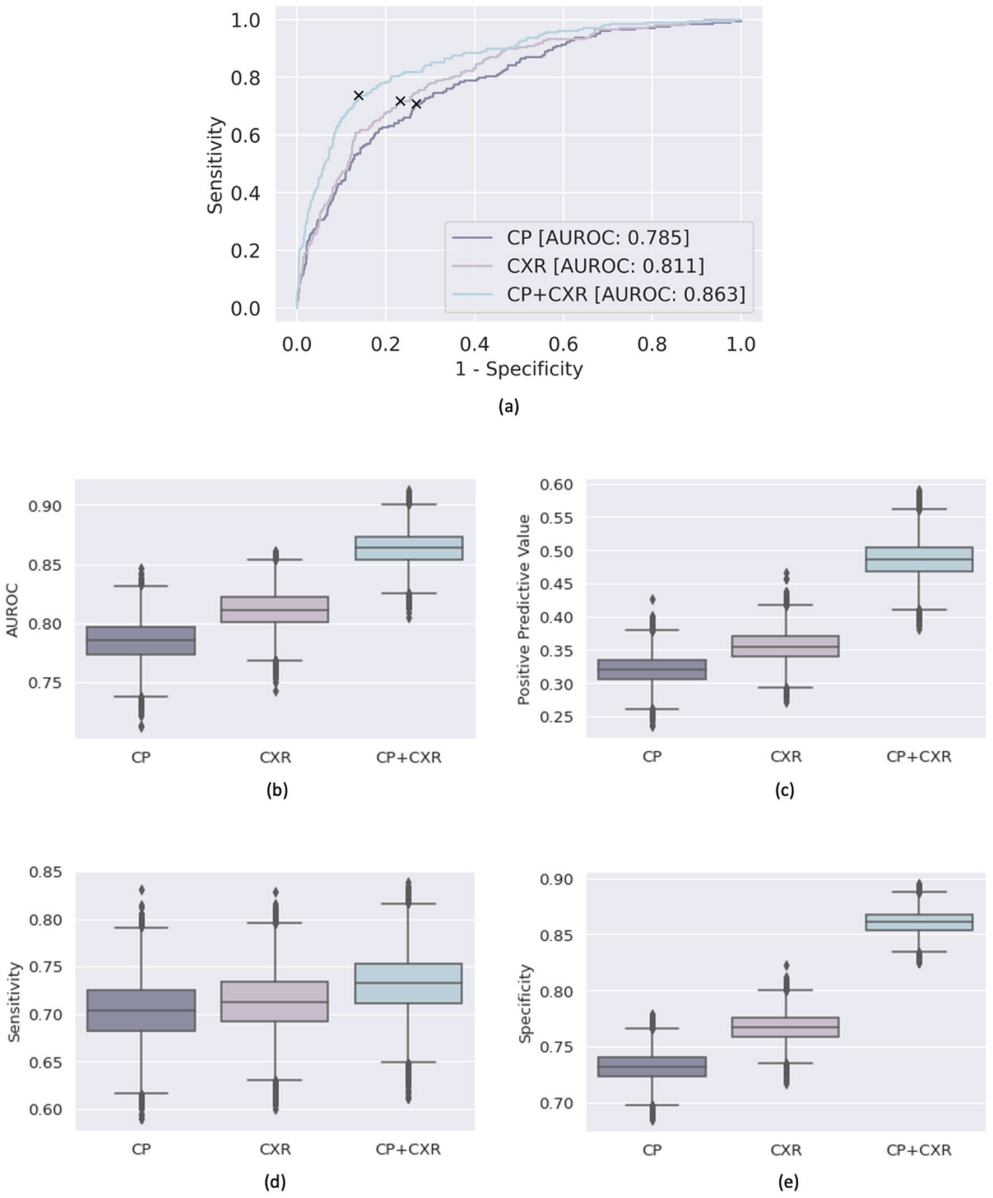
Detailed performance metrics of the Medical Transformer (MeTra). MeTra was trained on the clinical parameters only (CP), on the chest radiographs only (CXR), and the combined multimodal data (CP + CXR). Receiver operating characteristic (ROC) curves (**a**) and areas under the ROC curves (**b**). To determine discrimination thresholds, the operating point was determined by maximizing Youden’s criterion (sensitivity + specificity), resulting in specific values for the positive predictive value (**c**), sensitivity (**d**), and specificity (**e**). The combined model performed superior to the uni-modal models for every metric.

**Figure 3:**
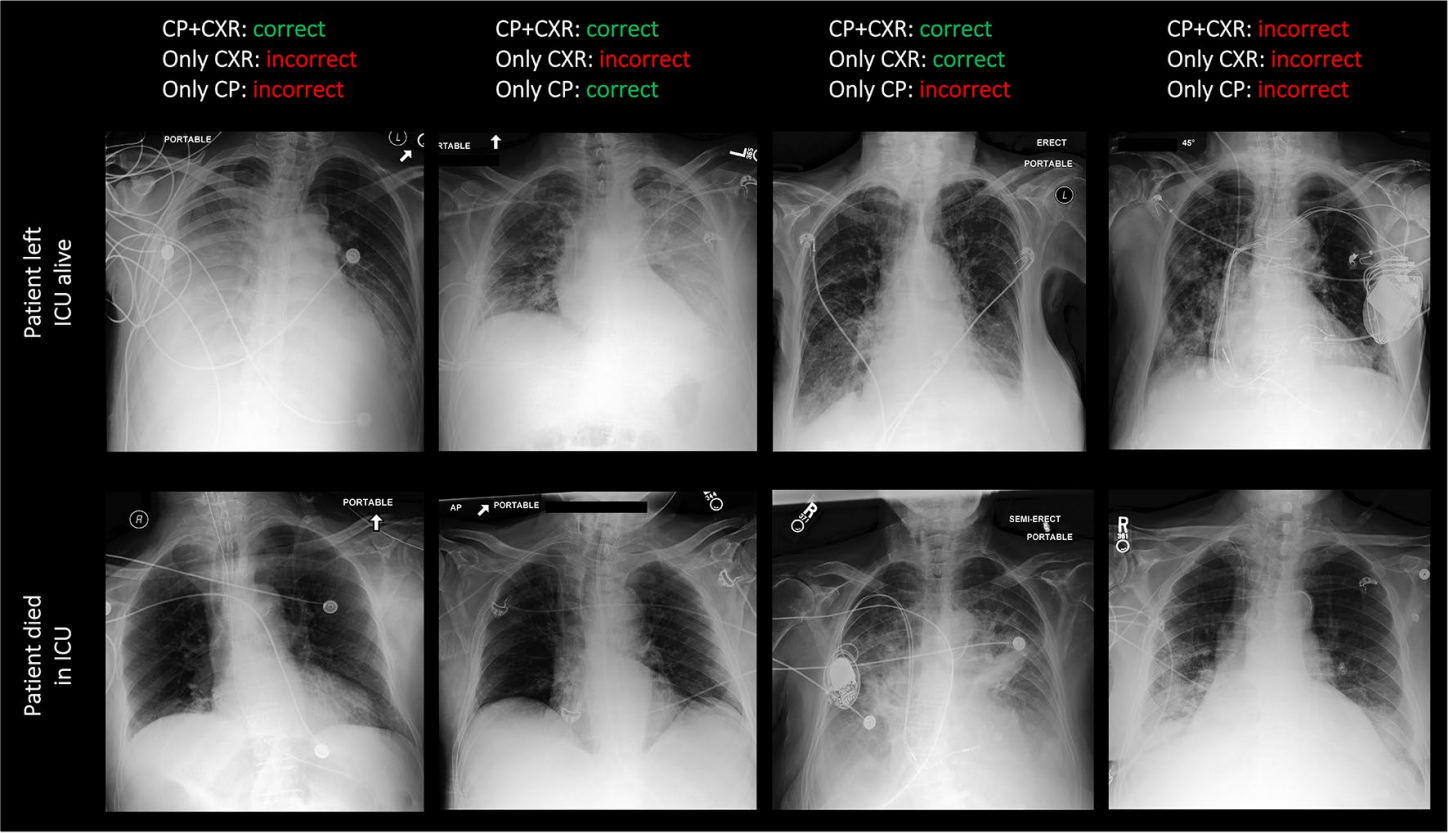
Exemplary chest radiographs and associated patient survival predictions. The upper row shows chest radiographs of four patients dismissed from the ICU alive. The lower row shows chest radiographs of four patients who died in intensive care. Predictions of the model were  or  depending on whether all data, i.e., imaging and non-imaging data (“CP + CXR”) was provided, or whether only the imaging data (“Only CXR”) or only the clinical parameters (“Only CP”) were provided. Please refer to Fig. 2 for an explanation of the abbreviations.

Table 2 and Fig. 2 summarize the MeTra model’s performance when trained on single data categories. When trained on 15 clinical parameters only, MeTra was characterized by an AUROC (area under the receiver operating characteristic curve) value of 0.785 [95% CI [confidence interval] 0.751, 0.819], a sensitivity of 0.703 [0.640, 0.766], a specificity of 0.731 [0.706, 0.756], and a positive predictive value of 0.320 [0.278, 0.363]. When trained on the chest radiographs only, MeTra reached an AUROC value of 0.811 [0.779, 0.841], a sensitivity of 0.713 [0.650, 0.773], a specificity of 0.767 [0.743, 0.791], and a positive predictive value of 0.355 [0.310, 0.401]. In all metrics, training on chest radiographs only tended towards better performance than training on clinical parameters only. Nevertheless, statistical significance was only found for specificity (p = 0.02), while the other statistical measures were not significantly different (AUROC, p = 0.14; sensitivity, p = 0.41; positive predictive value, p = 0.14). Exemplary images for correct and incorrect model predictions are given in Fig. 3. By trend, the combined model could correctly predict survival even when the unimodal models were contradictory in their predictions, e.g., when the radiograph was largely inconspicuous. Variable pulmonary opacifications and pleural effusions were noted in false negative and false positive predictions. Additional results can be found in Supplementary Fig. S1.

**Table 2:** Overview of the clinical parameters used in conjunction with the chest radiographs.

Variable	Type	Missing (%)	Mean (± std)	Impute value
Capillary refill rate	Categorical	100	–	–
Diastolic blood pressure	Continuous	0.04	59.04 (8.87) mmHg	59.0 mmHg
Fraction inspired oxygen	Continuous	26	0.45 (0.07) FiO2	0.21 FiO2
Glasgow coma scale—eye opening	Categorical	0	3.51 (0.66)	4
Glasgow coma scale—motor response	Categorical	0	5.13 (1.52)	6
Glasgow coma scale—verbal response	Categorical	0	4.35 (1.16)	5
Glasgow coma scale—total	Categorical	100	–	–
Glucose	Continuous	0.02	128.98 (28.72) mg/dL	128.0 mg/dL
Heart rate	Continuous	0	85.15 (12.96) bpm	86 bpm
Body height	Continuous	97.7	169.77 (9.00) cm	170.0 cm
Mean blood pressure	Continuous	0	74.15 (8.99) mmHg	77.0 mmHg
Oxygen saturation	Continuous	0	97.69 (1.98) %	98.0%
Respiratory rate	Continuous	0	18.95 (3.73) breaths per minute	19 breaths per minute
Systolic blood pressure	Continuous	0.04	113.87 (14.21) mmHg	118.0 mmHg
Temperature	Continuous	2.28	36.90 (0.32) °C	36.6 °C
Body weight	Continuous	9.13	79.73 (15.05) kg	81.0 kg
pH	Continuous	13.86	7.37 (0.06)	7.4

The column “Missing (%)” denotes the percentage of samples in the dataset that did not have any entry for this item.

### MeTra can be trained on multimodal data

When trained on both chest radiographs and clinical parameters, MeTra reached an AUROC value of 0.863 [0.835, 0.889], which was superior to both unimodal training settings (p < 0.001). Similarly, specificity (0.861 [0.841, 0.880], p < 0.001) and positive predictive value (0.486 [0.432, 0.541], p < 0.001) were significantly higher after multimodal training than after unimodal training (Fig. 2). Sensitivity was higher, too, yet not statistically significant (0.732 [0.670, 0.792], vs. unimodal_(chest radiographs only)_ = 0.33, vs. unimodal_(clinical parameters only)_ = 0.26).

### MeTra can deal with missing data

**Figure 4:**
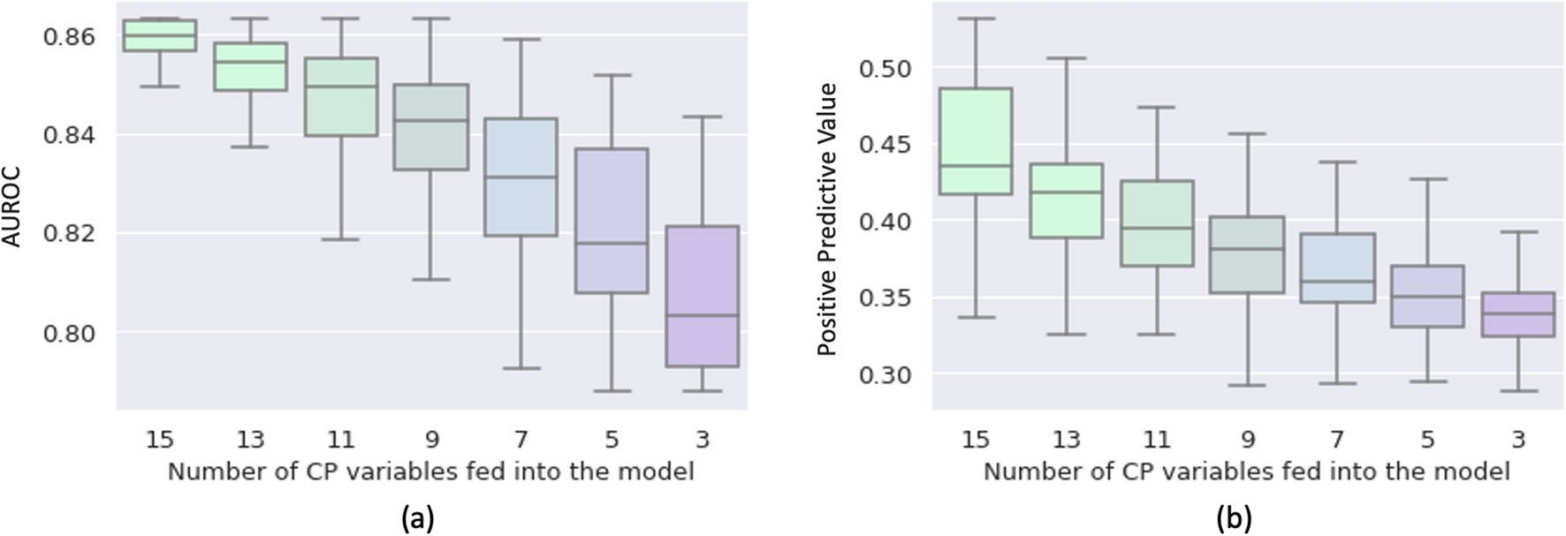
Performance of MeTra in terms of the AUROC values (**a**) and the positive predictive values (**b**) as a function of the number of clinical parameters available to the model. The x-axis denotes the number of clinical parameters fed into the model alongside the chest radiograph. For each number of clinical parameters, the experiment was repeated 100 times with randomly chosen subsets of variables to prevent bias due to the choice of variables.

The MeTra model can deal with missing data. However, like a physician with less data, MeTra’s predictions become less accurate when the number of available clinical parameters is reduced. For AUROC and the positive predictive values, a close-to-linear decrease is demonstrated as a function of reduced parameter availability (Fig. 4). Intentionally, we included the clinical parameters Glasgow Coma Scale (total) and capillary refill rate even though their content was empty for all the test samples. The upheld performance demonstrates robustness to the fact that labels might be missing a priori.

## Discussion

In this work, we developed and evaluated the medical transformer architecture MeTra to integrate imaging and non-imaging data for survival predictions in patients in critical care. While MeTra can predict the survival of critically ill patients when trained on clinical data or imaging data exclusively, the model can combine both data sources for improved model predictions. We also demonstrate that MeTra can deal with missing data and that there is a smooth transition between high diagnostic accuracy when all data is available to reduced diagnostic accuracy when data are missing. Consequently, MeTra may be considered a blueprint for how to utilize multimodal medical data in AI models.

Other groups have worked on survival prediction without transformer architectures and only achieved comparable performance when training on considerably more data and using extensive hyperparameter tuning (Table 3). The present study is the first to investigate the performance of a fully transformer-based architecture in the survival prediction of patients in intensive care and proves its viability when handling imaging and non-imaging data. However, alternative transformer-based approaches have been introduced to the medical domain. Zheng et al. used the attention mechanism of transformers in combination with a graph-based method to model patient relations and utilize modality-specific data^22^. Our study distinguishes itself by eliminating the need for more complex fusion mechanisms. Song et al. used transformers to combine optical coherence tomography images and visual field exams to diagnose glaucoma^23^. The data had to be presented in matrix view, which allowed the authors to tailor their architecture to the available format. The authors also resorted to a CNN for feature extraction prior to employing the transformer for modality fusion. This approach seems unsuitable for our clinical question that aims to combine non-imaging data, such as laboratory values (typically not available in matrix view), with imaging data. Moreover, using an additional CNN does not align with our objective of implementing a purely transformer-based model. Nguyen et al. introduced the CLIMAT (Clinically-Inspired Multi-Agent Transformers) model as a fully-transformer-based model for predicting the progression of knee osteoarthritis using imaging and non-imaging data^24^. The authors used three distinct transformer modules to (i) extract features from imaging data, (ii) extract features from non-imaging data, and (iii) combine the extracted features to provide a set of output predictions, where each corresponds to the disease severity at a specific point in time. While conceptionally, the authors followed a similar approach in using transformer blocks exclusively, the different clinical question necessitates architectural distinctions. In the CLIMAT model, multiple class tokens are added to the last transformer module to extract predictions for multiple time steps. Furthermore, a compressed representation of the non-imaging features is used and concatenated to each output token of the imaging-specific transformer module before the tokens are fed to the final transformer module. In contrast, we intentionally did not compile the non-imaging data before the multimodal data fusion to ensure that all information is visible to the model. Moreover, to make sure that each imaging token attends to all non-imaging tokens and vice versa, we feed the joint set of features as tokens through the last transformer module.

**Table 3:** Comparison of MeTra to current state-of-the-art methods for survival prediction in patients in intensive care in terms of area under the receiver operating characteristic curve (AUROC) and the area under the precision recall curve (AUPRC).

Method	AUROC	AUPRC	Comments
Early^14^	0.827 [0.801, 0.854]	0.485 [0.417, 0.555]	Clinical parameters (CP) and imaging data are first pre-trained separately. Subsequently, the latent representation of both modalities is concatenated, and a final classification layer is trained to merge the inputs
Joint^14^	0.825 [0.798, 0.853]	0.506 [0.436, 0.574]	CP and imaging data are fed through separate feature extraction layers and then concatenated and fed through a final classification head to form the final prediction. Training is performed in an end-to-end setting
MMTM^14,42^	0.819 [0.788, 0.846]	0.474 [0.402, 0.544]	A Multimodal Transfer Module^14,42^ (MMTM) is added after feature extraction of both modalities to merge the inputs
DAFT^14,16^	0.828 [0.799, 0.854]	0.492 [0.427, 0.572]	A Dynamic Affine Feature Map Transform^14,16^ (DAFT) is used after feature extraction of both modalities to scale and shift the resulting feature maps to merge the modalities
Unified^14,15^	0.835 [0.808, 0.861]	0.495 [0.424, 0.567]	In every training iteration, a two-step approach is performed. First, feature extractors for the CP and imaging data (which do not necessarily have to be paired) are trained separately to extract meaningful features. Second, the previously learned feature extractors extract features for a set of paired samples, which are then concatenated and fed through a learnable classification head
MedFuse (PT)^14^	0.841 [0.813, 0.868]	0.544 [0.477, 0.609]	For CP and imaging data, separate feature extractors are learned on modality-specific labels. The final prediction is then formed by feeding both feature representations sequentially into a neural network of LSTM (Long Short-Term Memory) layers This AUROC cannot directly be compared to our method: The configuration of MedFuse(PT) uses considerably more imaging data (pre-training on 340,470 additional radiographs) and more CP (22,356 samples) as compared to MeTra (6,798 samples for both CP and imaging data)
MedFuse (OPTIMAL)^14^	0.865 [0.837, 0.889]	0.594 [0.526, 0.655]	For CP and imaging data, separate feature extractors are learned on modality-specific labels. The final prediction is then formed by feeding both feature representations sequentially into a neural network of LSTM layers This AUROC cannot directly be compared to our method: MedFuse(OPTIMAL) uses the same additional imaging data as MedFuse(PT) and performs extensive selection on the CP data based on 22,356 samples
MedFuse (RI)^14^	0.817 [0.785, 0.846]	0.471 [0.404, 0.545]	For CP and imaging data, separate feature extractors are learned on modality-specific labels. The final prediction is then formed by feeding both feature representations sequentially into a neural network of LSTM layers This AUROC cannot directly be compared to our method: MedFuse (RI) is not pre-trained on additional imaging data (as PT or OPTIMAL) but still uses more CP data (22,356 samples) as compared to MeTra (6,798 samples)
MeTra (CP + CXR)	0.863 [0.835, 0.889]	0.594 [0.526, 0.662]	MeTra is based on the transformer model, where data is processed as a set of tokens. The CP and imaging data are fed through corresponding transformer-based backbones to extract latent feature tokens merged in a final transformer encoder MeTra is trained on fewer data than MedFuse OPTIMAL, PT, and RI

Means [95% confidence intervals].

Beyond, our work is clinically and scientifically relevant in several aspects:

First, our clinical experience teaches us that any predictive model used clinically must deal with missing data. Not all patients are treated and diagnosed equally, and the diagnostic toolset—from imaging to laboratory studies to clinical tests—is not consistently applied to all patients. The resultant data inconsistency and scarcity are problems for conventional machine learning models since the number of patients with “complete” datasets for training is inherently limited. MeTra solves this problem as it can both be *trained* on incomplete data and can also deal with missing data during *inference*.

Second, medical diagnosis is based on data from various sources: Medical doctors assess radiographs in conjunction with laboratory values, clinical tests, and history findings, among others. Developing machine learning models that rival human expertise will eventually require including data from all these sources. MeTra suggests one possible path forward by providing an architecture encompassing data from any source. Flexible data integration into the model is a beneficial feature of the transformer architecture that contrasts with other state-of-the-art network architectures such as CNNs. CNNs are specifically designed to work well on images and -even though possible- including non-imaging data remains challenging^25,26^.

Third, an improved survival prediction in intensive care can help assess illness severity and direct intensive care where needed to save lives and improve outcomes^3^. As detailed above, MeTra achieves state-of-the-art performance in this task. It may support physicians in clinical decision-making once clinical applicability beyond this proof-of-concept study has been demonstrated. We make the trained model open-source to facilitate future translational research efforts. For full transparency and comparability, we used the identical training test splits as others^14^, and this information is published with the MeTra model itself.

Previous research has utilized ensembles of conventional machine learning algorithms^3^, CNNs in conjunction with attention mechanisms^27^, or recurrent neural networks^14^ to predict patient survival. By comparison, the transformer architecture employed in MeTra has several advantages: It employs the same backbone architecture as the Vision Transformer^12^ and upholds its advantages in incorporating global information at shallow layers while being more robust to adversarial attacks than CNNs^28^.

Our work has limitations: first, the survival prediction and validation data originate from a single center due to the unique availability of imaging and non-imaging data alongside survival data. Consequently, no external validation was performed, and the model’s generalizability remains to be confirmed using multimodal datasets from other institutions and through other researchers. However, we hope our work stimulates collective efforts to assemble comparable large-scale databases. Perspectively, collective work on transformer models may be accelerated further by decentralized peer-to-peer collaborations, for example, using a swarm learning approach^29^. Second, we only included relatively basic physiologic measures used for patient monitoring, while more complex measures of hemodynamics, oxygen metabolism, and microcirculation were not considered. Third, because the number of deaths in the ICU was unbalanced, the resultant class imbalance is an issue that needs consideration. Future work may address the class imbalance during training, for example, by including a weight factor into the loss function (accounting for the class imbalance) or by oversampling the underrepresented class^30^. Additionally, a hybrid approach of transformer layers and a CNN backbone may be used to further improve the performance^31^. A more comprehensive analysis of hyperparameter choices could also be performed, e.g., the choice of vision dropout. Future studies should investigate the association between specific vision dropout settings and model performance. Fourth, the clinical dataset had missing data, and any imputation may introduce bias, increase the variability of the model’s performance, and affect the results. On scientific grounds, we intentionally used the same (inconsistent) impute values as other groups to compare our MeTra model to their models. A more systematic approach would benefit and result in more robust models. On clinical grounds, a thorough analysis of the model’s performance regarding missing and spurious data is required before deployment and use in the clinic. Specifically, excluding clinical parameter values by zero-tokens may lead to distribution shifts and impaired prediction performance. While we account for the distribution shifts through dropout layers in the model architecture of MeTra, future work should explore alternative methods to exclude zero-masked tokens from the input (for example, as introduced by He et al.^32^). Adopting their approach would involve masking out missing clinical events at specific time points that are fed into the model individually. However, the computational burden caused by the quadratic scaling and associated memory requirements should be considered. Fifth, when interpreting our results in the context of the pertinent literature, it is essential to realize that the referenced results of other groups’ models only indicate the range of potential outcomes. A more thorough comparison would require strict standardization of all aspects, i.e., the models would have to be trained on the same data, and the data processing pipeline would have to be identical with a fixed random seed for augmentations. Sixth, another limitation relates to the variable time difference between imaging and non-imaging data. The non-imaging (clinical) data were collected during the first 48 h after a patient had been admitted to the ICU. In contrast, the last chest radiograph acquired during a patient's ICU stay was included as the (paired) imaging data^14^. In the patient subpopulation of the MIMIC dataset that was included in our study (for whom clinical parameters and chest radiographs were available), patients had an average ICU stay length of 5.4 ± 4.9 d (range 1.1–99.6 d [n = 6125 patients]). In our clinical experience, ICU stay lengths are affected by admission diagnosis, patient demographics, constitution, comorbidities, complications, type of treatment, and others, which affect the variability of associated clinical parameters. Consequently, the substantial time difference outlined above is worth considering when drawing clinical conclusions. For any meaningful clinical insights, more specific clinical questions need to be asked, more refined patient populations need to be studied, and more fine-granular analyses need to be conducted. In addition, mortality may be determined by a range of conditions with limited bearings on the chest radiograph, which is inherently limited in differentiating pathologic processes characterized by similar radiographic changes, e.g., pulmonary opacifications^33^. In the clinic, the availability of clinical parameters aids in interpreting equivocal findings on chest radiographs and vice versa. Therefore, our findings of significantly improved survival predictions based on imaging and non-imaging data become clinically plausible, yet the real clinical benefit remains to be determined.

In conclusion, we developed and validated a multimodal medical transformer model that can be easily trained without specifically tweaking the architecture for specific input modalities and exhibits robustness to missing and heterogeneous data. We achieved excellent performance in the survival prediction of patients in critical care. We also make our model an open source for clinicians and researchers as a benchmark model on a well-defined dataset.

## Online methods

### Study design

Following approval by the local ethical committee (Reference No. 028/19), this retrospective study followed local data protection regulations. All networks were trained on publicly available datasets described below and tested for their performance in predicting the survival of patients in intensive care.

### Description of dataset

The MIMIC-IV (Medical Information Mart for Intensive Care) dataset is a large US database of retrospectively collected data from two in-hospital database systems: a custom hospital-wide EHR and an ICU-specific clinical information system. The MIMIC-IV dataset contains EHR data and is linked to the MIMIC-chest-X ray (MIMIC-CXR) database, which provides the corresponding imaging data of the same patients^21,34^. All data is publicly available via physionet^35^. For full transparency and optimal comparability, we have used the same training test splits as other groups^14^, and we publish this split alongside the model. Table 1 provides a detailed description of the dataset.

### Data preprocessing

The imaging and non-imaging data were extracted from the MIMIC database and preprocessed as described by Hayat et al.^14^ (Fig. 1). In detail, a subset of the MIMIC data was compiled, containing millions of clinical events corresponding to 17 clinical parameters (Table 2). Of these, the capillary refill rate and Glasgow Coma Scale (total) were missing for all patients and, thus, disregarded from our analysis, leaving 15 clinical parameters to be included in the model. The chest radiographs (obtained as anterior–posterior projections) from the MIMIC-CXR database were extracted and matched to the EHR data. The chest radiographs were first normalized to match the dataset statistics of ImageNet^36^ (in terms of means and standard deviations) and resized to a resolution of 384 × 384 to use pre-trained models (see below). Data were split into training (72%), validation (8%), and test (20%) data using patient-wise stratification but otherwise random allocation.

### The multimodal medical transformer architecture

**Figure 5:**
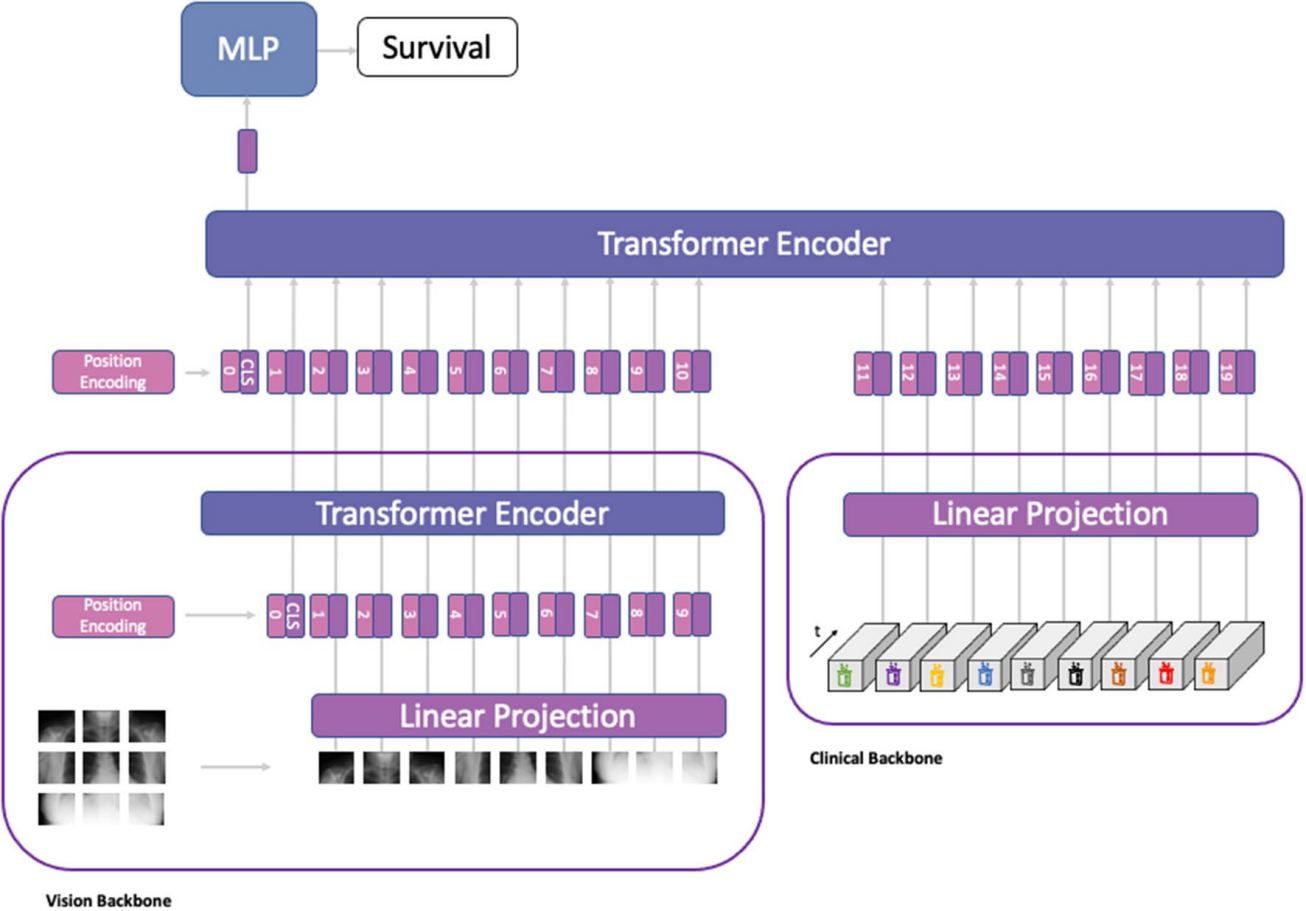
Medical Transformer (MeTra) architecture. The chest radiograph is first processed in the vision backbone, where it is split into patch embeddings and subsequently fed through a transformer encoder. Similarly, the clinical parameter items are fed through the clinical backbone, where they are projected to an embedding space with a dimensionality that matches that of the image embeddings. In the next step, a learnable position encoding token is added to the embeddings of both modalities. Finally, the modalities are fused by processing the embeddings with a transformer encoder that applies multi-head self-attention to all input tokens, thus allowing cross-modality information transfer. A multilayer-perceptron (MLP) is applied to the output to form the final prediction for in-hospital survival.

Building on the transformer architecture proposed by Vaswani et al.^13^, which was subsequently extended for use in vision problems^12^, we designed our medical transformer model to provide a direct way to incorporate imaging and non-imaging data into the learning process. Principally, as data inside transformer models is processed in tokens, there are no restrictions for its application on other modalities. More precisely, MeTra takes input data from two different modalities. Chest radiographs $x_{C \times R} \in {\mathbf{\mathbb{R}}}^{H \times W}$ of image height *H* and width *W* were first processed by a vision backbone to extract high-level image features $z_{C \times R} \in {\mathbf{\mathbb{R}}}^{N \times D}$ that could be fused with the data of other modalities later. Here, *N* denotes the number of tokens and *D* denotes the dimensionality of the latent representation for each token. Any vision transformer model can be used for this task, thus allowing us to leverage models pre-trained on different datasets. In particular, MeTra uses a Vision Transformer (ViT)^12^ with a patch size of 16 that has been pre-trained on ImageNet without the final classification head as its backbone. Additionally, clinical parameters retrieved from the EHRs $x_{CP} \in {\mathbf{\mathbb{R}}}^{K \times T}$ are projected into the latent representation $z_{CP} \in {\mathbf{\mathbb{R}}}^{M \times D}$ using a linear layer to match the dimensionality *D* of the image tokens. Here, *K* denotes the number of EHR items and *T* denotes the number of recorded time steps for each item. We set *T* to 48 in all experiments, representing the values of the respective item for each hour within the first 48 h of patient admission to the ICU. A missing value is imputed by setting it to the most recent measurement value if available or by setting it to a pre-specified value (Table 2) as suggested by Harutyunyan et al.^37^. To fuse imaging and non-imaging data efficiently, the latent representations of both backbones are concatenated to form the latent representation $z_{MULTI} \in {\mathbf{\mathbb{R}}}^{(N + M) \times D}$. The self-attention mechanism used inside transformers to process the input sequence does not consider the order of the elements in the sequence. To address this issue, we define a set of *N* + *M* learnable tokens of dimension *D* that are added element-wise to the latent representation $z_{MULTI}$. Subsequently, a learnable class token *CLS* is prepended to $z_{MULTI}$, and the resulting multimodal representation is processed with a transformer encoder, where the multi-head self-attention layers^13^ allow cross-modality information transfers. A multi-layer perceptron with a Sigmoid activation function is applied to the output to form the final prediction $p_{SURVIVAL}$ that quantifies the likelihood of in-hospital survival of the patient. The MeTra architecture is visualized in Fig. 5.

We trained three variants to compare the different modalities’ influence on the models’ final performance. The model only using the clinical parameters as retrieved from the EHR (“clinical parameters only-model”) was restricted to this source of data by setting the pixel values of the corresponding chest radiograph $x_{C \times R}$ to zero. Similarly, for the corresponding model that only used the chest radiographs for predictions (“radiographs-only model”), the clinical parameters $x_{CP}$ were set to zero. Finally, the combined model was trained by resuming the training routine from the checkpoint of the clinical parameters only-model with the highest area under the receiver operating characteristic curve (AUROC) value on the validation set (which is different from the test set). Motivated by preliminary findings [not shown] that indicated severe disbalance in the model’s focus and substantial disregard of the non-imaging data when trained on imaging and non-imaging data at once, we modified the training strategy of the combined model as follows: The imaging information was excluded during initial training and only provided (alongside the non-imaging information) during the subsequent training steps. Consequently, the combined model uses a similar setting as the unimodal models, i.e., starting from the same initial random states, but applying a full dropout of the imaging information during the initial epochs of training. No further restrictions on the available data were made; therefore, all information present in $x_{C \times R}$ and $x_{CP}$ were used. To further prevent the multimodal transformer encoder from relying exclusively on information originating from the vision backbone, all pixels in $x_{C \times R}$ were randomly set to zero with probability $p_{VDO}$ (chosen to be 30% and based on preliminary studies). We coined this procedure *vision dropout*.

The training was performed on an NVIDIA Quadro RTX 6000 for 200 epochs to guarantee the convergence of each model. As the learning objective, we minimized the binary cross-entropy loss:

$$L_{BCE} = y\cdot\log (p_{SURVIVAL} ) + (1 - y) \cdot \log (1 - p_{SURVIVAL} ),$$

where $y \in \{ 0,1\}$ represents the ground truth value for survival. 1 denotes that the patient died during the hospital stay, and 0 denotes that the patient was discharged alive. We used the AdamW^38^ optimizer with a learning rate of 5e − 6, which was decreased over time using the cosine annealing procedure^39^ until a final learning rate of 1e − 7 was reached. The entire code was written using Python v3.8, and MeTra was implemented using PyTorch v1.11.0. For more information regarding our training procedure, please refer to Supplementary Table S1.

### Description of experiments

In the first experiment, the model was trained only on the clinical parameters and subsequently evaluated with these data as exclusive input.

In the second experiment, the model was trained only on the imaging data and evaluated with these data only.

In the third experiment, the model was trained on all data and evaluated using all data.

The combined model (third experiment) was provided with the full imaging data set but only parts of the clinical parameters as input to study how missing data impact its performance. In detail, this experiment was repeated 100 times with 2, 4, 6, 8, 10, 12, and 14 clinical parameters set to “missing” each time. Missing parameters were chosen randomly within each of the 100 runs to prevent bias in choosing variables.

We evaluated the AUROC, AUPRC, sensitivity, specificity, and positive predictive value for all experiments.

### Statistical analysis

Statistical analyses were conducted using Python v3.8 with its libraries *NumPy* and *SciPy*. Bootstrapping was employed with 10,000 redraws for each measure to determine the statistical spread and calculate p-values for differences^40^. For calculating sensitivity and specificity, a threshold was chosen according to Youden’s criterion^41^, i.e., a threshold that maximized (sensitivity + specificity). We included all patients for which both radiographs and clinical parameters were available.

## Supplementary Information

Supplementary Information.

## Data Availability

All data, including imaging and non-imaging data, is publicly available from the MIMIC database^21,34^ on PhysioNet (for MIMIC-IV, see https://physionet.org/content/mimiciv/1.0/. and for MIMIC-CXR-JPG, see https://physionet.org/content/mimic-cxr-jpg/2.0.0/). The code to extract the chest radiographs and corresponding clinical parameters can be found in the GitHub repository linked in the code availability section.

## Code availability

The entire code is publicly available on GitHub via https://github.com/FirasGit/MeTra. We also provide detailed information on all data splits into training and testing for other groups to compare their algorithms with ours.
