# Supplementary material for: Medical transformer for multimodal survival prediction in intensive care: integration of imaging and non-imaging data
Source: Sci Rep. 2023 Jul 1;13:10666. doi: 10.1038/s41598-023-37835-1 (PMC10314902; doi:10.1038/s41598-023-37835-1)
Supplement: Supplementary file 1 — Supplementary Information. [file 41598_2023_37835_MOESM1_ESM.docx]

Medical Transformer for Multimodal Survival Prediction in Intensive Care - Integration of Imaging and Non-Imaging Data

Authors: Firas Khader* M.Sc. (1)., Jakob Nikolas Kather Prof. (2, 3, 4, 5), Gustav Müller-Franzes M.Sc. (1), Tianci Wang B.Sc. (1), Tianyu Han M.Sc. (6), Soroosh Tayebi Arasteh M.Sc. (1), Karim Hamesch MD (2), Keno Bressem MD (7), Christoph Haarburger M.Sc. (8), Johannes Stegmaier Prof. (9), Christiane Kuhl Prof. (1), Sven Nebelung^†^ MD (1), Daniel Truhn^†^ MD (1)

#

# Supplemental Material

**Supplementary Table 1:** Hyperparameters used for training our Medical Transformer (MeTra) neural network architecture. All models (CP, CXR, CP+CXR) are trained with the same set of hyperparameters. Please refer to **Figure 2** for an explanation of the abbreviations.

| Batch size | 50 |
| --- | --- |
| Learning Rate | 5e-6 |
| Scheduler | Cosine Annealing^38^ |
| Optimizer | AdamW^37^ |
| Epochs | 200 |
| Image Size | 384 x 384 |
| Image augmentations (only applied to training set) | - Horizontal flipping   ([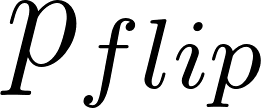](https://www.codecogs.com/eqnedit.php?latex=p_%7Bflip%7D#0) = 50%)  - Rotations (±45°) - Scaling (± 15%)  - Translation (± 15%) |
| Vision Dropout probability [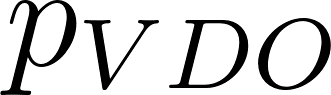](https://www.codecogs.com/eqnedit.php?latex=p_%7BVDO%7D#0) | 30% |
| **Vision Backbone** |  |
| - Patch size | 16 x 16 |
| - Embedding dimensionality | 384 |
| - Depth | 12 |
| - Number of heads | 6 |
| **Clinical Backbone** |  |
| - Embedding dimensionality | 384 |
| **Transformer Encoder (final)** |  |
| - Embedding dimensionality | 384 |
| - Depth | 4 |
| - Number of heads | 4 |


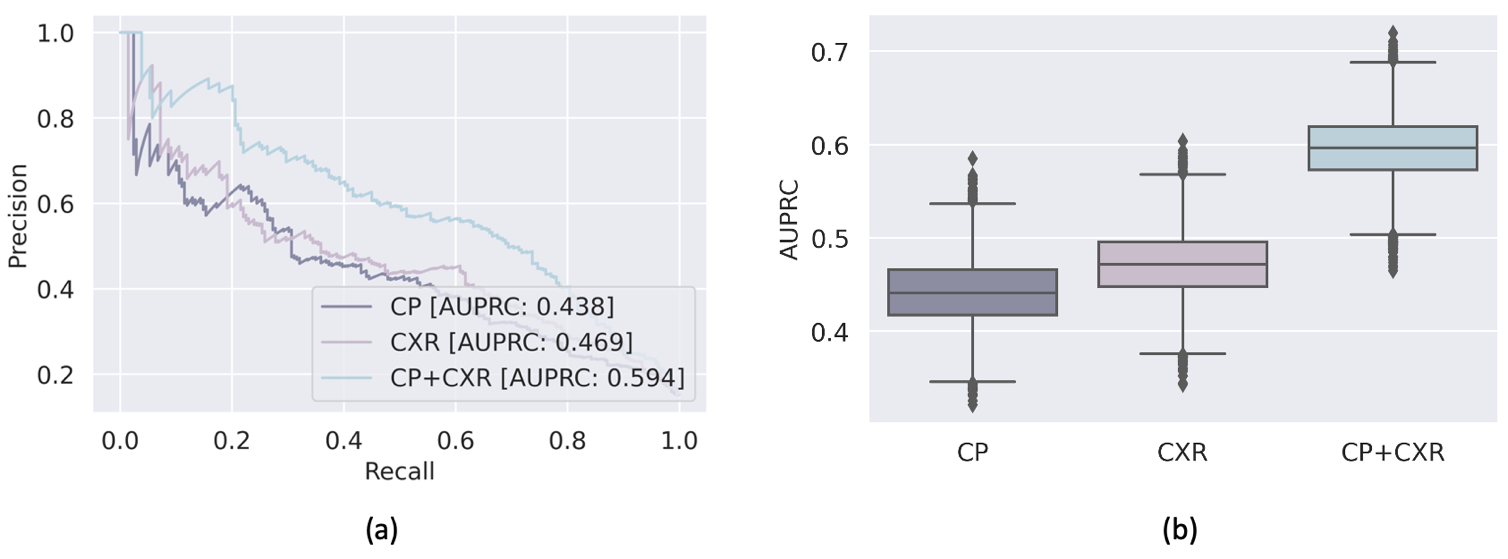
 **Supplementary Figure 1**: Performance of the Medical Transformer (MeTra) architecture trained on the clinical parameters only (CP), on the chest radiographs only (CXR), and the combined multimodal data (CP+CXR). Precision recall curve (a) and areas under the precision recall curve (AUPRC) (b).
